# Portal Hyperperfusion after Extended Hepatectomy Does Not Induce a Hepatic Arterial Buffer Response (HABR) but Impairs Mitochondrial Redox State and Hepatocellular Oxygenation

**DOI:** 10.1371/journal.pone.0141877

**Published:** 2015-11-02

**Authors:** Stefan Dold, Sven Richter, Otto Kollmar, Maximilian von Heesen, Claudia Scheuer, Matthias W. Laschke, Brigitte Vollmar, Martin K. Schilling, Michael D. Menger

**Affiliations:** 1 Department of General-, Visceral-, Vascular- and Pediatric Surgery, University of Saarland, Homburg/Saar, Germany; 2 Institute for Clinical & Experimental Surgery, University of Saarland, Homburg/Saar, Germany; 3 Institute for Experimental Surgery, University of Rostock, Rostock, Germany; IDIBAPS - Hospital Clinic de Barcelona, SPAIN

## Abstract

**Background & Aims:**

Portal hyperperfusion after extended hepatectomy or small-for-size liver transplantation may induce organ dysfunction and failure. The underlying mechanisms, however, are still not completely understood. Herein, we analysed whether hepatectomy-associated portal hyperperfusion induces a hepatic arterial buffer response, i.e., an adaptive hepatic arterial constriction, which may cause hepatocellular hypoxia and organ dysfunction.

**Methods:**

Sprague-Dawley rats underwent 30%, 70% and 90% hepatectomy. Baseline measurements before hepatectomy served as controls. Hepatic arterial and portal venous flows were analysed by ultrasonic flow measurement. Microvascular blood flow and mitochondrial redox state were determined by intravital fluorescence microscopy. Hepatic tissue pO2 was analysed by polarographic techniques. Hepatic function and integrity were studied by bromosulfophthalein bile excretion and liver histology.

**Results:**

Portal blood flow was 2- to 4-fold increased after 70% and 90% hepatectomy. This, however, did not provoke a hepatic arterial buffer response. Nonetheless, portal hyperperfusion and constant hepatic arterial blood flow were associated with a reduced mitochondrial redox state and a decreased hepatic tissue pO2 after 70% and 90% hepatectomy. Microvascular blood flow increased significantly after hepatectomy and functional sinusoidal density was found only slightly reduced. Major hepatectomy further induced a 2- to 3-fold increase of bile flow. This was associated with a 2-fold increase of bromosulfophthalein excretion.

**Conclusions:**

Portal hyperperfusion after extended hepatectomy does not induce a hepatic arterial buffer response but reduces mitochondrial redox state and hepatocellular oxygenation. This is not due to a deterioration of microvascular perfusion, but rather due to a relative hypermetabolism of the remnant liver after major resection.

## Introduction

Partial- or reduced-size-liver transplantation has been established to counterbalance the lack of donor organs in liver transplantation [1]. Moreover, extended liver resection is a widely used procedure to treat hepatocellular carcinoma and colorectal liver metastases. These procedures, however, are associated with distinct portal hyperperfusion, which is thought to cause postoperative organ dysfunction, resulting in small-for-size or small-for-flow syndrome (SFSS) [2–4].

Liver perfusion is characterized by an unique self-regulating mechanism, known as hepatic arterial buffer response (HABR) [5,6] The HABR describes the ability of the hepatic artery to react on changes in portal venous blood flow. A decreased portal blood flow leads to an increased hepatic arterial flow [7]. This provides the maintenance of organ perfusion, function and homeostasis as well as a constant clearance of hormones and drugs. Lautt et al. indicated that the HABR is caused by an adenosine wash-out [8]. According to this, adenosine is secreted constantly in the so-called space of Mall, a microscopic fluid compartment, which surrounds the portal triad in the periportal part of the liver acinus. Thus, a decreased blood flow in the portal vein reduces the adenosine wash-out, resulting in a vasodilatation of the hepatic artery. The p1-purinoceptor-subtype A2 could be identified as effector structure to mediate this adenosine-induced vasodilatation [9].

Portal hyperperfusion after small-for-size liver transplantation or extended liver resection may cause a decrease of hepatic arterial blood flow representing a HABR. Inversely to the theory of Lautt, portal hyperperfusion should result in an increased adenosine wash-out and, consequently, to a constriction of the hepatic artery associated with a reduced blood flow. After small-for-size liver transplantation, several studies have indeed indicated a reduction of hepatic arterial blood flow [10,11]. Little is known, however, whether portal hyperperfusion after hepatectomy induces a HABR. Previous studies have indicated a slight but not significant reduction of hepatic arterial flow per wet weight after two third hepatectomy in rats [12–14]. Whether a more extended hepatectomy is capable of inducing a HABR is not known. The aim of the present study was therefore to elucidate which extent of hepatectomy results in a diminished hepatic arterial perfusion and whether this is associated with hepatic microcirculatory disorders, hepatocellular hypoxia and organ dysfunction.

## Materials and Methods

### Animals

Adult Sprague-Dawley rats (body weight (bw): 357±31g) of either sex were used for the experiments. Animals were housed one per cage on a 12h/12h light-dark cycle and had free access to standard pellet food and tap water ad libitum. The experiments were conducted in accordance with the German legislation on protection of animals and the National Institutes of Health Guide for the Care and Use of Laboratory Animals (Institute of Laboratory Animal Resources, National Research Council NIH publication 86–23, revised 1985). The experiments were approved by the local animal care committee (Department V, safety and regulation division, district police department 66424 Homburg, Germany) under reference number 29/2004.

### Surgical procedure

Rats were anesthetized with pentobarbital sodium (50mg/kg bw, intraperitoneally, Narcoren; Braun, Melsungen, Germany) and tracheotomized to facilitate spontaneous breathing. The animals were then placed in a supine position on a heating pad, maintaining body temperature at 36–37°C. A catheter (PE-50, 0.58 mm inner diameter (i.d.); Portex, Hythe, UK) was placed in the right carotid artery for continuous monitoring of mean arterial blood pressure and heart rate, fluid substitution (3mL/h saline), and blood sampling to determine arterial blood oxygenation (automated blood gas analyser Rapidlab 800, Bayer HealthCare LLC, East Walpole, USA). After opening the abdominal cavity by midline incision the hepatic artery, the portal vein and the common bile duct were gently separated from the hepatoduodenal ligament. Common bile duct was punctured for insertion of a catheter (PE-30, 0.28mm i.d.; Portex, Hythe, UK) to analyse bile flow and biliary bromosulfophthalein (BSP) excretion.

### Experimental protocol

#### Liver resection

For microvascular and hepatic functional analyses rats (n = 8) underwent step by step hepatectomy. After a 30-min baseline monitoring, the first step was a 30% hepatic resection (left lateral liver lobe). In a second step further 40% of the liver mass was removed (median liver lobe), corresponding to an overall 70% resection. Finally, further 20% of liver tissue was removed (right superior and inferior liver lobe), resulting in an overall 90% hepatectomy [15]. After each step a 60-min period was provided. During the second half of each of this period ultrasonic flow measurements, intravital fluorescence microscopy, bile volume analysis and partial oxygen pressure monitoring in arterial blood and liver tissue were performed. Baseline analysis before hepatectomy served as control measurements.

#### Biliary BSP excretion

In additional experiments biliary BSP excretion was analysed in animals without hepatectomy (n = 5) and after 90% hepatectomy (n = 5).

#### Selective hepatic clamping

To confirm the results of ultrasonic flow measurements, additional animals (n = 5) underwent instead of hepatectomy a selective clamping of 70% of liver tissue mass (right medial, left lateral and left medial liver lobe) by placing a microvascular clip on the left and median branches of the hepatic artery, the portal vein and the bile duct. After induction of anaesthesia, instrumentation of the animal and laparotomy, a steady state period of 30 min was used to perform the in vivo measurements. Then a 70% clamping was induced and in vivo measurements were performed at 30 min to 60 min after induction of clamping. The measurements included the determination of portal venous and hepatic arterial blood flow.

At the end of each operation rats were sacrificed by an overdose of pentobarbital sodium (100mg, intravenously, Narcoren; Braun, Melsungen, Germany).

### Ultrasonic flow measurements

An ultrasonic perivascular flow probe (0.5V; Transonic Systems, Ithaca, NY, USA) was placed around the hepatic artery. A second flow probe (1.5R; Transonic Systems) was positioned around the portal vein. This experimental approach allowed the simultaneous assessment of hepatic arterial and portal venous blood flow. The flow probes were connected to a flow meter (T206 Animal Research Flowmeter, Transonic Systems) and blood flow was recorded over 5 minutes at each time point.

### Hepatic tissue oxygenation

Partial oxygen pressure (pO_2_) of the liver tissue was assessed by means of a flexible polyethylene microcatheter Clark type pO_2_ probe (diameter, 470 μm; length, 300 mm) (LICOX System; GMS, Kiel-Melkendorf, Germany). Online temperature compensation was performed by an additional temperature probe (type K thermocouple probe, LICOX System).

### Intravital fluorescence microscopy

Before and after each step of hepatectomy, the hepatic microcirculation was analysed by intravital fluorescence microscopy. Therefore, a Zeiss Axiotech microscope (Zeiss, Oberkochen, Germany) and an epi-illumination technique with a 100 W mercury lamp was used [16,17]. Microscopic images were registered by a charge coupled device (CCD) video camera (FK 6990 B-IQ, COHU; Prospective Measurements Inc., San Diego, CA, USA) and transferred to a video system (S-VHS Panasonic AG 7350-E, Matsushita, Tokyo, Japan) for subsequent off-line analysis. Sodium fluorescein (2 mmol/kg bw; Merck, Darmstadt, Germany) was injected for contrast enhancement to visualize sinusoidal perfusion using blue light epi-illumination (450–490/>520nm excitation/emission wavelength) [18]. Hepatocellular NADH fluorescence was analysed as an indicator of the mitochondrial redox state using ultraviolet epi-illumination (330 to 380/>415nm) [19].

Assessment of hepatic microcirculatory parameters was performed off-line by frame-to-frame analysis of the videotaped images using a computer-assisted image analysis system (CapImage; Zeintl, Heidelberg, Germany). Within 10 lobules per animal, functional sinusoidal density was determined by counting the number of perfused sinusoids crossing a 200 μm raster line [17,20]. For estimation of volumetric blood flow, red blood cell (RBC) velocity and the respective vessel diameters were measured within 7–10 individual sinusoids and 5 postsinusoidal venules per animal [7]. Volumetric blood flow (VQ) in the individual microvessels was estimated from RBC velocity and microvascular cross-sectional area according to VQ = RBC velocity*π*r^2^. The wall shear rate was calculated as an indicator for intravascular mechanical forces, which may affect the endothelial lining of hepatic microvessels. Values of wall shear rate (φ) were calculated based on the Newtonian definition: φ = 8*V/D, where V represents the RBC velocity of the individual microvessel and D the respective diameter. Densitometric analysis of hepatocellular NADH autofluorescence was performed in 5–7 midzonal regions by grey-level determination as described previously in detail [19].

### Bile and BSP excretion

In order to quantify ATP-dependent hepatic bile excretion 1mg/kg b.w. BSP was injected in the carotid artery. After 20 minutes the volume of bile collected was determined gravimetrically by assuming a density of 1g/mL and the bile concentrations of BSP and its conjugates were measured spectrophotometrically at the absorption maximum (580nm) after appropriate dilution with 0.05 M NaOH [21].

### Histology

Tissue samples were harvested after each step of hepatectomy from the resected liver lobes, fixed in formalin (4% in phosphate-buffered saline) for 2–3 days at 4°C and embedded in paraffin. Dehydrated, paraffin-embedded 5 μm sections were stained with hematoxylin and eosin (H&E) and analysed under light microscopy.

### Immunohistochemistry

To study apoptotic cell death by immunohistochemistry, specimens were incubated overnight at room temperature with a rabbit polyclonal antibody against cleaved caspase-3 (1:50; Cell Signaling Technology, Frankfurt, Germany). The antibody detects endogenous levels of the large fragment (17/19kDa) of activated caspase-3, but not full-length caspase-3. A biotinylated anti-rabbit immunoglobulin antibody was used as a secondary antibody for streptavidin-biotincomplex peroxidase staining (Link, LSAB-HRP; DakoCytomation, Hamburg, Germany). 3,3-diaminobenzidine was used as chromogen. The sections were counterstained with hemalaun. Quantitative analysis was performed by counting the number of positively stained cells, and data are given as positive cells per high power field.

### Western blot analysis

In additional animals a 30%, 70% hepatectomy or 90% hepatectomy was performed and liver tissue was harvested for Western blot analysis at 3 h after hepatectomy. Non-hepatectomized animals served as controls. For whole protein extracts and Western blot analysis of endothelial nitric oxide synthase (eNOS), phosphorylated eNOS (p-eNOS), inducible nitric oxide synthase (iNOS) and in addition hemeoxygenase-1 (HO-1), manganese superoxide dismutase 2 (SOD2), hypoxia-inducible factor-1α (HIF-1α) as well as adenosine and adenosine A_2a_ receptor (A_2A_R), liver tissue was homogenized in lysis buffer (10 mM Tris, pH 7.5, 10 mM NaCl, 0.1 mM EDTA, 0.5% Triton X-100, 0.02 mM NaN_3_, 0.2 mM PMSF, and protease inhibitor cocktail 1:100 vol/vol; Sigma-Aldrich, Taufkirchen, Germany). After incubation for 30 min on ice, samples were centrifuged for 30 min at 16,000 *xg*. The supernatant was saved as whole protein extract fraction. Protein concentrations were determined by using the Lowry assay with bovine serum albumin as standard. Fifteen micrograms of protein per lane were separated discontinuously on 10% SDS-PAGE gels and were transferred to polyvinylidene difluoride membranes (0.2 μm, Bio-Rad, Munich, Germany). After blockade of nonspecific binding sites, membranes were incubated for 4 h with a rabbit polyclonal anti-rat eNOS antibody (1:200, BD Biosciences, Heidelberg, Germany), a rabbit polyclonal anti-rat iNOS antibody (1:200, Abcam, Cambridge, UK), a rabbit polyclonal anti-rat p-eNOS antibody (1:300, Cell Signaling Technologies, by New England Biolabs GmbH, Frankfurt, Germany), a rabbit polyclonal anti-rat HO-1 antibody (1:500, Enzo Life Sciences GmbH, Lörrach, Germany), a mouse monoclonal anti-rat SOD2 antibody (1:100, E10, Santa Cruz Biotechnology, Heidelberg, Germany), a rabbit monoclonal anti-rat HIF-1α antibody (1:200, Abcam), a rabbit polyclonal anti-rat adenosine antibody (1:200, BioVision, Milpitas, CA, USA) or a mouse monoclonal anti-rat A_2A_R antibody (1:100, H82, Santa Cruz biotechnology). This was followed by a 1.5h-incubation with the corresponding peroxidase-conjugated secondary antibodies (1:5000, GE Healthcare, Freiburg, Germany). Protein expression was visualized by means of luminol enhanced chemiluminescence (ECL, GE Healthcare) and exposure of the membranes to a blue light-sensitive autoradiography film (Hyperfilm, GE Healthcare). Signals were densitometrically assessed (Gel Doc, Quantity One software; Bio-Rad) and were normalized to β-actin signals (mouse monoclonal anti- β-actin antibody, Sigma-Aldrich) to correct for unequal loading.

### Statistics

All data are presented as mean values ± SEM. After testing for normality and equal variance across groups, differences between the groups were assessed by one-way ANOVA, followed by an appropriate post hoc analysis, including Bonferroni probabilities to compensate for multiple comparisons. If normality or equal variance test failed, statistical analyses were performed using Kruskal-Wallis one-way ANOVA on ranks followed by multiple comparisons versus control group (Dunn’s method) (SigmaStat; Jandel Corporation, San Rafael, CA, USA). Statistical significance was accepted for a value of p<0.05.

## Results

### Systemic hemodynamics

No significant differences in heart rate between the groups could be observed. However, mean arterial pressure was significantly (p<0.05) decreased after 90% hepatectomy (85 ± 5 mmHg) when compared with that measured at baseline (114 ± 4 mmHg) and after 30% hepatectomy (119 ± 4 mmHg).

### Hepatic blood flow

**Fig 1:**
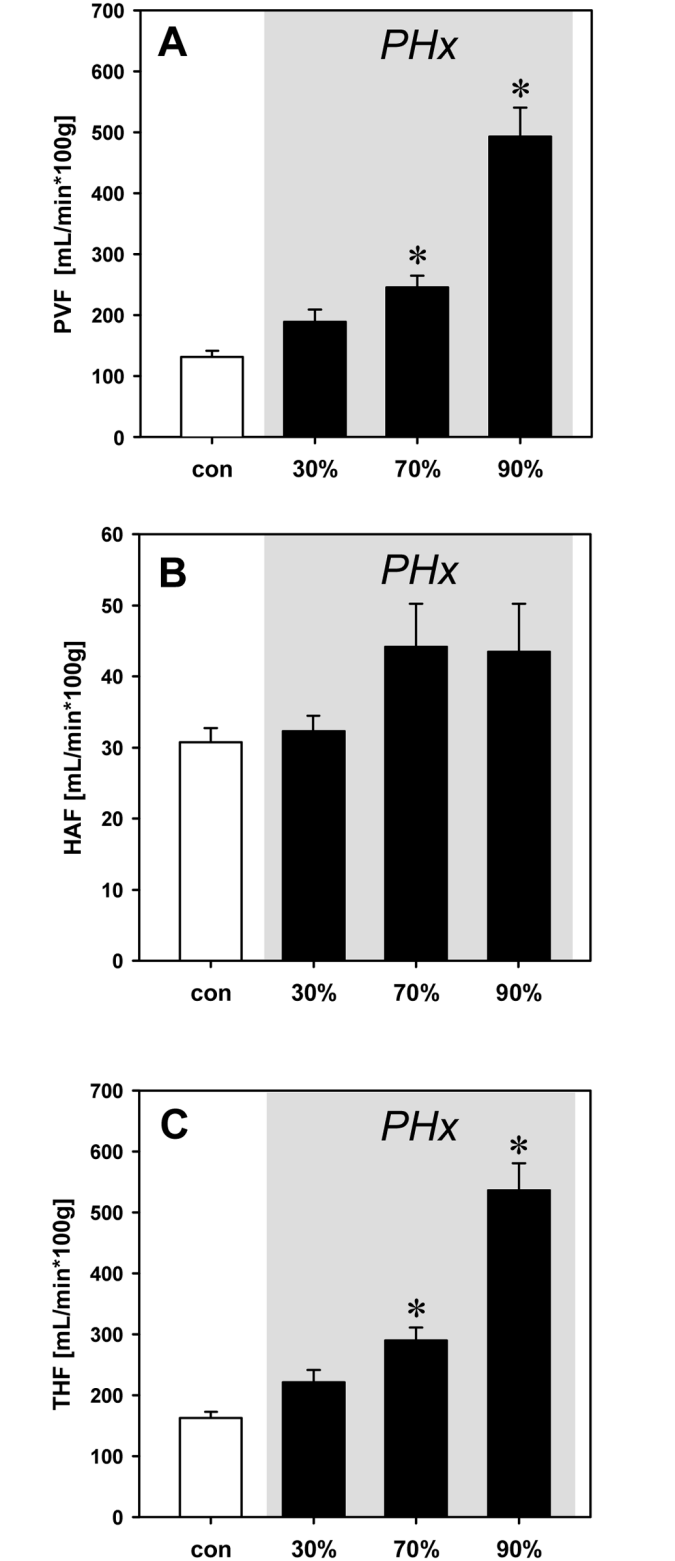
Hepatic blood flow. Portal venous blood flow (PVF; A), hepatic arterial blood flow (HAF; B), and total hepatic blood flow (THF; C) per 100g liver wet weight under baseline conditions (con) and after 30%, 70% and 90% hepatectomy (PHx). Flow measurements were performed with ultrasonic flow probes. Mean values ± SEM, *p<0.05 vs. con.

Hepatectomy resulted in a distinct portal hyperperfusion. This was indicated by a massively increased blood flow in the portal vein after 70% and, in particular, after 90% hepatectomy when compared to baseline. In contrast, hepatic arterial blood flow did not significantly change throughout the experiment, indicating the lack of a HABR. As a consequence, 70% liver resection resulted in a 2-fold increased total hepatic blood flow and 90% resection provoked an almost 4-fold increase of liver perfusion (Fig 1).

Accordingly, selective clamping of the median and left lateral liver lobes resulted in a comparable portal hyperperfusion of the liver tissue (279 ± 23% of baseline). Of interest, the hepatic arterial blood flow showed no significant changes after clamping (115 ± 17%), confirming the findings obtained after hepatectomy.

### Hepatic microcirculation

Diameters of liver sinusoids and postsinusoidal venules were significantly increased after both 70% and 90% hepatectomy. Also, RBC-velocity in liver sinusoids and postsinusoidal venules was found elevated after these two major resection procedures (Table 1). Accordingly, the blood flow in the hepatic sinusoids and postsinusoidal venules was increased, in particular after 90% hepatectomy (Table 1). In parallel, the wall shear rate was significantly elevated after 90% hepatectomy in both sinusoids and postsinusoidal venules (Table 1). Of interest, functional sinusoidal density was found only slightly affected, presenting with a ~10% reduction after 90% hepatectomy (Table 1).

**Table 1:** Hepatic microcirculation after hepatectomy.

Extent of hepatectomy	con	30%	70%	90%
**Diameter sinusoids [μm]**	6.4 ± 0.3	6.8 ± 0.2	7.1 ± 0.4*	7.3 ± 0.3*
**Diameter venules [μm]**	25.3 ± 0.9	28.9 ± 1.8	30.1 ± 2.4*	31.4 ± 1.7*
**RBC velocity sinusoids [mm/s]**	0.2 ± 0.0	0.4 ± 0.0*	0.5 ± 0.1*	0.8 ± 0.0*
**RBC velocity venules [mm/s]**	0.8 ± 0.1	1.1 ± 0.1	1.4 ± 0.1*	3.6 ± 0.2*
**Sinusoidal blood flow [pL/s]**	7.3 ± 0.7	12.0 ± 1.8	20.3 ± 3.1*	35.2 ± 4.2*
**Venular blood flow [pL/s]**	415.0 ± 54.3	702.6 ± 156.0	980.9 ± 107.7	2904.0 ± 447.7*
**Sinusoidal shear rate [1/s]**	286.2 ± 21.4	435.7 ± 29.0	580.0 ± 65.0	916.3 ± 58.7*
**Venular shear rate [1/s]**	249.4 ± 19.3	314.1 ± 25.0	388.1 ± 56.3	928.6 ± 44.2*
**Sinusoidal density [n/mm]**	45 ± 1	44 ± 1	42 ± 2	40 ± 2*

Hepatic microcirculation after 30%, 70% and 90% hepatectomy. Baseline conditions without hepatectomy (con) served as controls. Means ± SEM.

*p<0.05 vs. con.

### Hepatic tissue oxygenation and mitochondrial redox state

**Fig 2:**
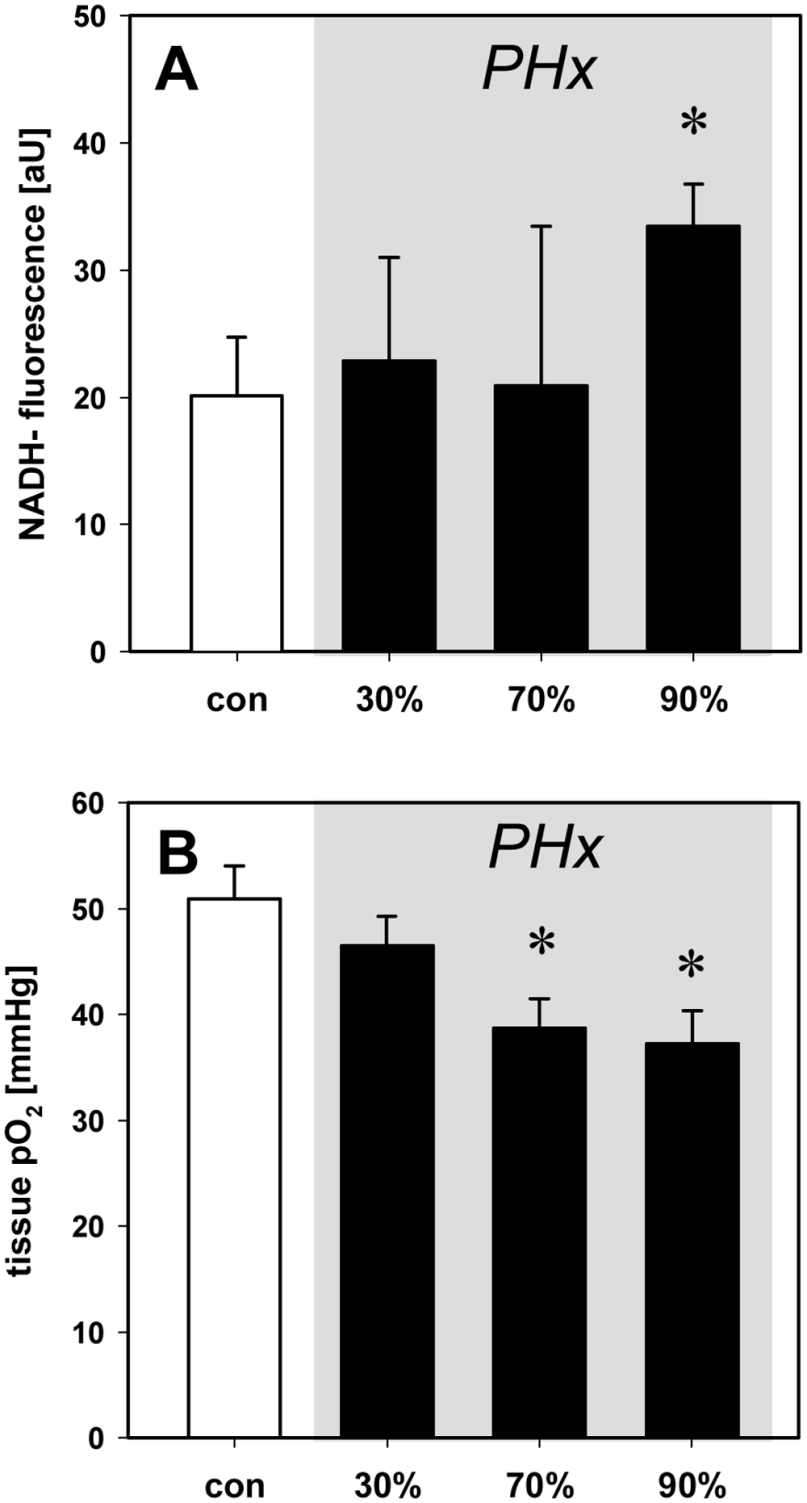
Hepatic tissue oxygenation and mitochondrial redox state. Hepatic tissue pO_2_ (A) and NADH fluorescence (B) under baseline conditions (con) and after 30%, 70% and 90% hepatectomy (PHx). Tissue pO2 was measured by polarographic techniques and NADH fluorescence was determined by intravital epi-illumination microscopy. Mean values ± SEM, *p<0.05 vs. con.

Despite portal hyperperfusion and unchanged hepatic arterial blood flow (Fig 1), hepatic tissue pO_2_ was significantly decreased after 70% and 90% hepatectomy (Fig 2). In line with these findings, 90% hepatectomy resulted also in an increase of NADH fluorescence when compared to baseline, indicating an impaired mitochondrial redox status of the liver tissue (Fig 2). Of interest, systemic arterial pO_2_ did not change over the entire experimental period (data not shown).

### Bile flow and BSP secretion

**Fig 3:**
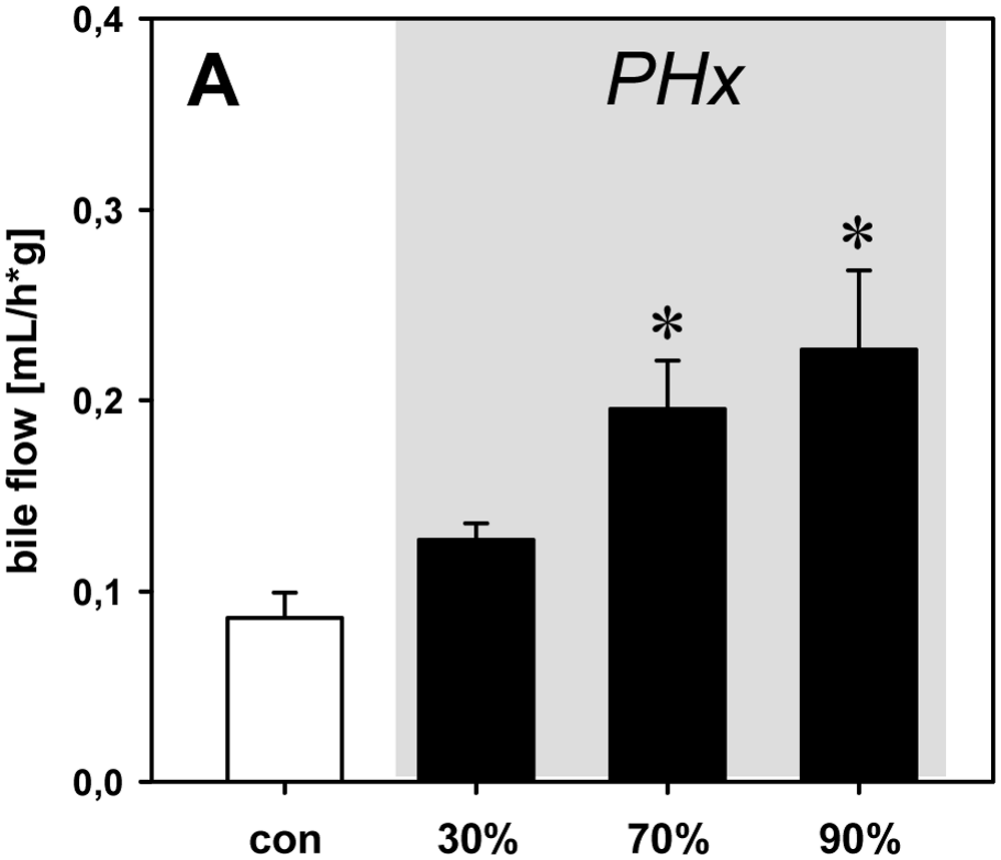
Bile flow. Bile flow per gram liver wet weight under baseline conditions (con) and after 30%, 70% and 90% partial hepatectomy (PHx). Mean values ± SEM, *p<0.05 vs. con.

Analysis of bile flow per gram liver wet weight showed a significant 2-3-fold increase after 70% and 90% hepatectomy when compared to baseline measurements (Fig 3). Analysis of active bile transport, i.e. BSP excretion, revealed a ~2-fold increase of BSP excretion after 90% hepatectomy (0.81 ± 0.34 mg/h*g) compared to non-hepatectomized controls (0.36 ± 0.06 mg/h*g).

### Tissue integrity

Analysis of H&E-stained liver sections did not indicate relevant signs of hypoxia-associated hepatocellular disintegration such as vacuolization in hepatocytes. Analysis of the number of caspase-3-positive cells, indicating apoptotic cell death, was below 1%, and was not found changed after 30%, 70% and 90% hepatectomy.

### Protein expression

**Fig 4:**
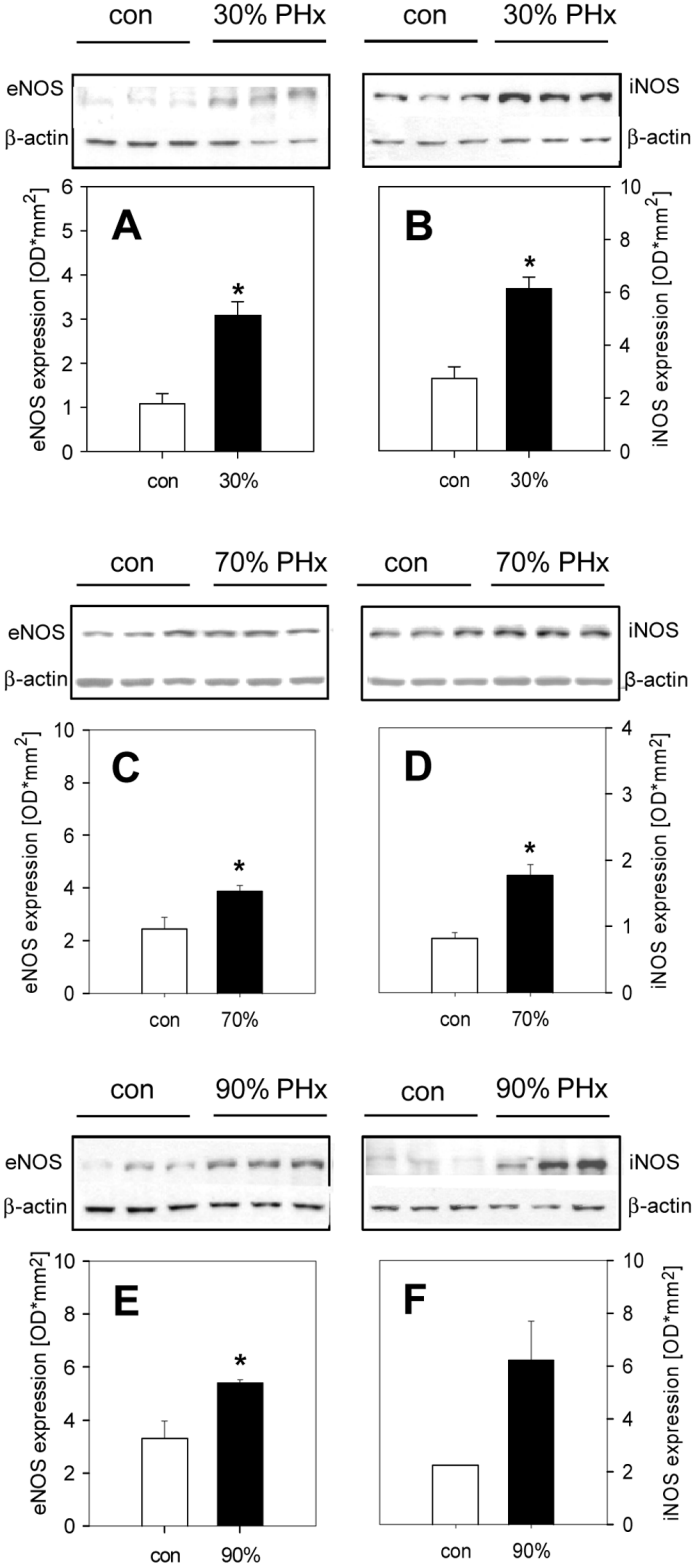
eNOS and iNOS expression. Protein expression of endothelial (eNOS, A,C,E) and inducible nitric oxide synthase (iNOS, B,D,F) before (con, n = 3) and 3 h after 30% (A,B), 70% (C,D) and 90% (E,F) hepatectomy (PHx, n = 3). Mean values ± SEM, *p˂0.05 vs. con.

**Fig 5:**
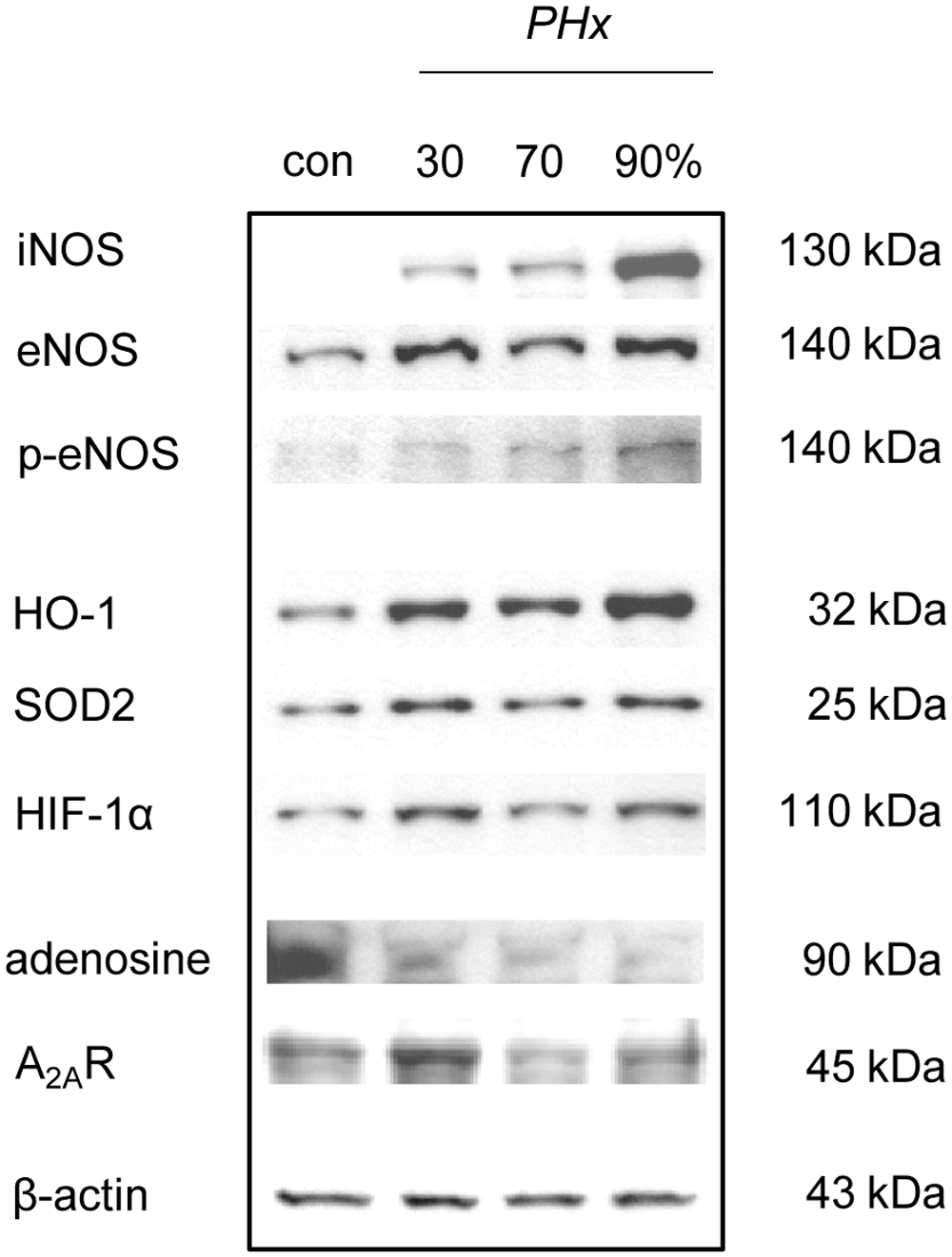
eNOS, iNOS, p-eNOS, HO-1, SOD2, HIF-1α, adenosine and A_2A_R expression. Representative western blot analyses of the protein expression of inducible (iNOS), endothelial (eNOS), phosphorylated endothelial nitric oxide synthase (p-eNOS), hemoxygenase-1 (HO-1), manganese superoxide dismutase 2 (SOD2), hypoxia-inducible factor-1α (HIF-1α) as well as adenosine and adenosine A_2a_ receptor (A_2A_R) before (con) and 3 h after 30%, 70% and 90% hepatectomy (PHx).

To study the role of nitric oxide in the regulation of liver perfusion after hepatectomy, protein expression of eNOS and iNOS was determined by Western blot analyses. Of interest, 30% as well as 70% and 90% hepatectomy resulted in a distinct increase of both eNOS and iNOS protein expression in the remnant liver tissue (Fig 4). In addition, phosphorylated eNOS was found also markedly increased after hepatectomy (Fig 5), resulting in an increase of the p-eNOS/eNOS ratio after 30% (0.52), 70% (0.95) and 90% hepatectomy (1.18) when compared to control (0.49). Of interest, this was associated with an increased expression of HIF-1α (Fig 5).

To study a potential role of adenosine, adenosine and A_2A_R expression were analyzed after hepatectomy. These analyses showed a marked decrease of adenosine after 30% hepatectomy, but in particular after 70% and 90% hepatectomy. In contrast, the expression of the A_2A_R was not found substantially changed after hepatectomy (Fig 5). To study a role of reactive oxygen species further analyses of indirect markers, including HO-1 and SOD2, were performed. These analyses showed an increased expression of both HO-1 and SOD2 after 30%, 70% and 90% hepatectomy when compared to control (Fig 5).

## Discussion

The major finding of the present study is that portal hyperperfusion neither after 30% nor after 70% and 90% hepatectomy induce a HABR, i.e. an adaptive hepatic arterial constriction. Of interest, however, the liver remnants present with a reduced mitochondrial redox state and a decreased hepatocellular oxygenation despite the overall hepatic hyperperfusion after extended hepatectomy. This is not due to a deterioration of hepatic microvascular perfusion, but rather due to a relative hypermetabolism of the remnant liver.

A hepatic arterial blood flow response upon portal hyperperfusion is still a matter of debate. Several reports have demonstrated that after orthotopic small-for-size liver transplantation portal hyperperfusion is associated with a significantly diminished hepatic arterial blood flow [10,11,22], indicating a HABR. The reduction of the hepatic arterial blood flow after small-for-size liver transplantation may, however, not be due to a portal hyperperfusion-induced HABR, but rather due to a pronounced increase of norepinephrine serum levels that can be measured immediately after transplantation. Hickman et al. postulated that norepinephrine should be considered as a substantial contributing factor inducing the arterial flow reduction in small-for-size grafts [22]. In line with this, Kostapanagiotou and co-workers showed that the hepatic arterial flow reduction in small-for-size liver transplants is associated with a persistent 6- to 8-fold elevation of norepinephrine serum concentrations [23]. These authors also showed that the maintenance of hepatic arterial blood flow after normal size liver transplantation is paralleled by normalized norepinephrine serum concentrations [23]. The reduced liver mass after small-for-size transplantation may cause an impaired extraction of plasma norepinephrine, resulting in persistently increased serum concentrations. Thus, the reduction of hepatic arterial blood flow after small-for-size liver transplantation may not be due to a portal hyperperfusion-induced increased adenosine wash-out, as would be expected for a HABR, but rather due to persistently increased norepinephrine levels.

In contrast to small-for-size liver transplantation, the results of the present study could not confirm a hepatic arterial flow reduction after extended hepatectomy, indicating an only minor role of norepinephrine during the postoperative period. This view is in line with results of others, demonstrating that partial hepatectomy, similar as sham operation, results in an only short-term increase of norepinephrine serum concentration with rapid normalization to baseline [24]. This difference in hepatic arterial blood flow response after hepatectomy compared to small-for-size transplantation may be the cause for the fact that in clinical practice extended liver resection up to 80% is well tolerated while the outcome after small-for-size transplantation is poor with grafts less than 50% of the recipients liver volume [2].

The lack of a HABR after major hepatectomy despite portal hyperperfusion may not necessarily disprove the theory of Lautt [25]. Additional factors such as nitric oxide (NO) synthases may be activated after hepatectomy, counteracting the hepatic arterial flow response. Macedo & Lautt could show that elevated shear stress within the vascular system induces NO release that suppresses arterial constriction [26]. In addition, there is a large body of evidence that hypoxia in various settings augments NO synthase expression and NO release [27,28]. The significant increase of hepatic microvascular shear stress and the marked decrease of liver tissue oxygenation, observed in the present study, suggest a major role of NO liberation in counteracting a HABR (hepatic arterial constriction) after extended hepatectomy. This is further supported by the increased expression of both eNOS and iNOS after major liver resection, as shown in the present study and also reported by others [29]. Of interest, the increased eNOS expression is most probably caused by the increased hyperperfusion-induced shear stress [30,31], while hypoxia and liver regeneration may be responsible for the increase of iNOS [32,33].

Beside the action of NO, an increased adenosine secretion may also be considered as a potential mechanism counteracting a portal hyperperfusion-induced hepatic arterial flow reduction. It is well known that hypoxia can increase adenosine release by inhibiting adenosine kinase [34]. Choukèr et al. could demonstrate a significant increase of adenosine serum levels under hypoxic conditions, and Cray et al. could further show that hypoxia-induced adenosine mediates an increase in hepatic arterial blood flow [35,36]. However, the decreased expression of adenosine, observed in the present study, does not support the view that unchanged hepatic arterial flow after hepatectomy is due to the vasodilatory action of hypoxia-induced adenosine, blunting the portal hyperperfusion-associated HABR.

The present study shows a significantly reduced liver tissue oxygenation and an impairment of the mitochondrial redox state after extended hepatectomy. This was observed despite portal hyperperfusion and an unchanged hepatic arterial blood flow. Thus, other mechanisms apart from hypoperfusion have to account for the deteriorated tissue oxygenation. These may include impaired oxygen uptake or increased hepatocellular oxygen consumption. It is well known that portal hyperperfusion is associated with an increased shear stress, which is capable of causing distinct endothelial damage, including cell swelling and detachment [37]. This could be the cause for impaired oxygen diffusion and, thus, a reduced uptake by the liver tissue. However, the present study indicates only minor alterations of the hepatic microcirculation. Sinusoidal perfusion was found increased independent of the extent of the hepatectomy and sinusoidal density was not affected after 30% and 70% hepatectomy, and only slightly affected after 90% liver resection. On the other hand, the present study shows that extended liver resection is associated with an elevated bile flow and an increased biliary BSP secretion. This is in line with previous studies, demonstrating an increased bile flow and bile acid output in the early prereplicative period of liver regeneration after 70% hepatectomy [38]. The increased liver function indicates an enhanced hepatocyte metabolism, which is associated with increased oxygen consumption. Thus, the impaired mitochondrial redox state and the decreased tissue oxygenation after extended hepatectomy despite portal hyperperfusion and unchanged hepatic arterial flow may not to be due to a deterioration of microvascular perfusion, but rather due to a relative hypermetabolism of the remnant liver.

## Abbreviations

SFSS: small-for-size syndrome
HABR: hepatic arterial buffer response
bw: body weight
i.d.: inner diameter
BSP: bromosulfophthalein
pO_2_: oxygen pressure
CCD: charge coupled device
RBC: red blood cell
VQ: volumetric blood flow
φ: shear rate
H&E: hematoxylin and eosin
eNOS: endothelial nitric oxide synthase
iNOS: inducible nitric oxide synthase
con: control
PVF: portal venous flow
HAF: hepatic arterial flow
THF: total hepatic flow
PHx: hepatectomy
